# How modelling can help steer the course set by the World Health Organization 2021-2030 roadmap on neglected tropical diseases

**DOI:** 10.12688/gatesopenres.13327.1

**Published:** 2021-07-27

**Authors:** Jessica Clark, Wilma A. Stolk, María-Gloria Basáñez, Luc E. Coffeng, Zulma M. Cucunubá, Matthew A. Dixon, Louise Dyson, Katie Hampson, Michael Marks, Graham F. Medley, Timothy M. Pollington, Joaquin M. Prada, Kat S. Rock, Henrik Salje, Jaspreet Toor, T. Déirdre Hollingsworth

**Affiliations:** 1 Big Data Institute, Li Ka Shing Centre for Health Information and Discovery, University of Oxford, Old Road Campus, Headington, Oxford, OX3 7LF, UK; 2 Institute of Biodiversity, Animal Health & Comparative Medicine, College of Medical, Veterinary & Life Sciences, University of Glasgow, Glasgow, G12 8QQ, UK; 3 Department of Public Health, Erasmus MC, University Medical Center Rotterdam, Rotterdam, 3000 CA, The Netherlands; 4 London Centre for Neglected Tropical Disease Research, Department of Infectious Disease Epidemiology, School of Public Health, Imperial College London, Norfolk Place, London, W2 1PG, UK; 5 MRC Centre for Global Infectious Disease Analysis, Department of Infectious Disease Epidemiology, School of Public Health, Imperial College London, Norfolk Place, London, W2 1PG, UK; 6 Schistosomiasis Control Initiative Foundation, London, SE11 5DP, UK; 7 Mathematics Institute, University of Warwick, Coventry, CV4 7AL, UK; 8 School of Life Sciences, University of Warwick, Coventry, CV4 7AL, UK; 9 Department of Clinical Research, London School of Hygiene & Tropical Medicine, London, WC1E 7HT, UK; 10 Faculty of Infectious and Tropical Diseases, London School of Hygiene & Tropical Medicine, London, WC1E 7HT, UK; 11 Centre for Mathematical Modelling of Infectious Disease, London School of Hygiene & Tropical Medicine, 15-17 Tavistock Place, London, WC1H 9SH, UK; 12 School of Veterinary Medicine, Faculty of Health and Medical Sciences, University of Surrey, Guildford, GU2 7AL, UK; 13 Department of Genetics, University of Cambridge, Cambridge, CB2 3EH, UK

**Keywords:** Mathematical, Statistical, Targets, Public Health, Elimination, Transmission, Cross-cutting, NTD

## Abstract

The World Health Organization recently launched its 2021-2030 roadmap, *Ending* *the* *Neglect* *to* *Attain* *the* *Sustainable Development Goals* *,* an updated call to arms to end the suffering caused by neglected tropical diseases. Modelling and quantitative analyses played a significant role in forming these latest goals. In this collection, we discuss the insights, the resulting recommendations and identified challenges of public health modelling for 13 of the target diseases: Chagas disease, dengue, *gambiense* human African trypanosomiasis (gHAT), lymphatic filariasis (LF), onchocerciasis, rabies, scabies, schistosomiasis, soil-transmitted helminthiases (STH), *Taenia solium* taeniasis/ cysticercosis, trachoma, visceral leishmaniasis (VL) and yaws. This piece reflects the three cross-cutting themes identified across the collection, regarding the contribution that modelling can make to timelines, programme design, drug development and clinical trials.

## Disclaimer

The views expressed in this article are those of the authors. Publication in Gates Open Research does not imply endorsement by the Gates Foundation.

## A renewed roadmap for a new decade

The World Health Organization’s (WHO) 2021-2030 Neglected Tropical Disease (NTD) Roadmap was launched on January 28 ^th^, 2021, renewing the commitment of the global NTD community to end the suffering caused by these diseases ^1^. The development of the roadmap was guided by extensive global stakeholder consultation, including consultation with mathematical and statistical modellers. Modellers were asked to assess the technical feasibility of proposed goals, to identify major challenges for achieving the new goals from a transmission dynamics perspective, possible acceleration strategies, and key outstanding research questions ^2^. Technical commentaries have been published as a collection in Gates Open Research ^3– 15^, which detail these insights for 13 NTDs: Chagas disease, dengue, *gambiense* human African trypanosomiasis (gHAT), lymphatic filariasis (LF), onchocerciasis, rabies, scabies, schistosomiasis, soil transmitted helminthiases (STH), *Taenia solium* taeniasis/ cysticercosis, trachoma, visceral leishmaniasis (VL) and yaws.

Neglected tropical diseases continue to affect over one billion people ^16^ as the result of the considerable inequalities in global healthcare systems that fail to support those most in need ^17^. The burden of NTDs falls largely on the poorest communities, resulting in an unrelenting cycle of poverty that is driven by negative social, health and economic impacts of infection on individuals and families, augmenting existing social divides. For infections with a substantial zoonotic component, morbidity and mortality among livestock also affect people’s livelihood with economic impacts that transcend medical implications. Notable progress to reduce the burden of NTDs has been made as a result of the commitments made in 2012 through the WHO 2020 NTD Roadmap ^18^ and the London Declaration on NTDs ^19^. As a result, 500 million people no longer require interventions against several NTDs and 40 countries, territories and areas have eliminated at least one disease ^1^. These wins are the outcome of concerted and consolidated efforts from endemic communities and invaluable volunteers, governments, donor agencies and the pharmaceutical industry. Despite such early gains, reaching the endgame presents some of the greatest challenges – namely sustaining those early gains whilst identifying and averting small numbers of sparsely distributed cases. The 2030 roadmap is shaped around three pillars that aim to support global efforts to maintain the gains, address the challenges and ultimately combat NTDs ^1^: 1. Accelerating programmatic action. 2. Intensifying cross-cutting approaches and 3. Shifting operating models and culture to facilitate in country ownership.

 The use of mathematical and statistical modelling in NTD research and policy has until recently, and with a few exceptions (e.g., onchocerciasis ^20^), lagged behind other groups of infectious diseases that receive more focus and funding (often, diseases that impact wealthier individuals and nations, or those perceived to potentially impact these). However, this is changing with the advent of groups like the NTD Modelling Consortium ^21^, who have developed the Policy-Relevant Items for Reporting Models in Epidemiology of Neglected Tropical Diseases (PRIME-NTD) principles, as a guide to communicate the quality and relevance of modelling to stakeholders ^20^. This has added clout to the call for modelling in the policy arena as well as setting a high bar of best practice for the wider modelling community. Having now gained significant traction, the use of modelling in NTD policy has contributed to new intervention tools ^22^, vector control strategies ^23– 26^, shaped policy responding to COVID-19-related programme disruptions ^27– 35^ and has aided in the development of WHO guidelines ^36, 37^. For this positive relationship to continue, it is imperative to invest in a mutual understanding through ongoing conversation between policy-makers and modellers, to determine what kind of questions are the “right” questions, how to interpret uncertainty and what the models can and cannot be used for.

This piece introduces a collection of papers borne of a meeting in Geneva, in April 2019 attended, among others, by the NTD Modelling Consortium and convened by the WHO: *Achieving NTD control, Elimination and Eradication Targets Post-2020; Modelling Perspectives & Priorities* ^2^. As new management targets and strategies took shape, the meeting provided policy makers and modellers the space to ask and answer specific questions regarding the proposed 2030 goals and the intended strategies to achieve them. Although the roadmap covers a range of diseases with diverse epidemiologies and differing management recommendations, the priority questions identified by modelers and stakeholders during the 2019 meeting and echoed by the authors of the technical commentaries shared three similar themes that should be considered in NTD modelling moving forward: timelines, programme design, and clinical study design. 

## Timelines

Goals are only worth setting in the context of time. It is therefore not surprising that many of the technical commentaries in this collection identified timelines as a priority issue. The public health and economic benefits of reaching goals are innumerable but can only be achieved by the target year through appropriate mobilisation of diverse resources. Modelling in the forms of past inference and forward projections can align many moving parts (for example epidemiological, demographic, and social considerations) to inform our understanding of the reasons why programmes succeed or fail ^38, 39^. Forecasts have played a crucial role in understanding whether the 2020 ^40^ and associated collection ^41, 42^, 2025 ^43^ and 2030 ^3– 15, 34^ goals can be reached under current strategies with the caveat that long-term predictions naturally become more uncertain.

In some instances, whether a goal can or will be met on time is relatively easy to ascertain – for example it is a resounding no for leprosy and rabies, which are hindered by passive case control, long quiescent incubation periods, and inadequate investment in interventions ^15, 44^. Alternatively, the goals for schistosomiasis ^11^, STH ^8^, and onchocerciasis ^13^ seem achievable in some or most settings, depending on localised parameters like baseline prevalence, and already experienced duration of and adherence to mass drug administration (MDA) programmes. In the case of *T. solium*, a lack of internationally agreed goals for elimination or control curtails the ability to effectively model timelines; for example, the 2021-2030 NTD roadmap proposes the overall milestone of achieving “intensified control in hyperendemic areas”, without agreeing on technical definitions for *T. solium* endemicity levels, or defining measurable criteria for attaining “intensified” control ^14^.

## Programme design

The diseases considered by the London Declaration and WHO roadmaps are at differing stages in their trajectories. Whilst some are on the cusp of achieving their goals, others face political and epidemiological barriers to progress. Both scenarios raise several priority questions regarding programme design, where ‘programme’ can mean intervention or surveillance. In addition to determining success or failure within the defined intervention time frames, modelling has provided insights into key factors of operational design like the treatment coverage necessary to reach goals in a given setting. Where it may not be possible, models can be used to test the efficacy of separate and combined chemotherapeutic ^37^ and non-pharmaceutical interventions ^23, 45, 46^, including combined interventions that target multi-host systems for zoonotic NTDs ^14^. Additionally, deciding the optimal timing ^47^ or frequency ^48, 49^ of treatment, and knowing who to treat ^50, 51^ are essential to the success of all interventions. Of course, the intervention strategies most likely to lead to achievement of the goals may not be sustainable in terms of cost to individuals, governments, or donors. By partnering highly detailed transmission models with cost-effectiveness analysis, modelling can also contribute to tailored insights regarding the affordability and benefits versus costs of interventions ^52– 62^. Models can also be used to explore integration between NTD programmes, or to understand the potential cross-utility of existing NTD programmes on other helminth species, such as exploring the additional benefit of national schistosomiasis control programmes using praziquantel on *T. solium* prevalence in co-endemic areas ^14^. Understanding this cross-utility is vital to intensifying cross-cutting approaches – one of the three core pillars of the roadmap, that differentiates the framework from its predecessor. 

These are all very practical features of intervention programmes that can in principle be planned for, but underlying features of target populations and human nature can undermine these plans. Survey data in recent years have made it evident that whilst the aim may be to deliver treatment at a certain geographical and therapeutic coverage, it is not analogous with consumption, as treatment is systematically not ingested by some ^63, 64^, or is not disseminated to the full intended group, reducing the true coverage. There are a variety of reasons for this ^65, 66^, but it is likely that similar mechanisms impact participation in surveillance, therefore biasing the estimates of prevalence, particularly when treatment and surveillance are co-occurring (e.g., gHAT ^9, 67^, rabies ^15, 68^). Modelling shows that the impact of this variable true coverage depends on the pathogen in question and transmission intensity ^64, 69– 71^ but it undoubtedly has an impact on reaching public health goals ^72, 73^.

Once a strategy has been deemed effective and prevalence targets are attained, it is likely that these interventions either transition, such as going from MDA to identified case management, or they stop all together. Establishing robust surveillance strategies at this point is vital, but obviously not everyone can be regularly sampled and not every incident infection case will be detected. Stochastic events like reinfection and reintroduction are risks that can drive resurgence. Modelling can support the identification of the optimal surveillance strategy and determine which prevalence or intensity indicators need to be monitored to ensure the desired public health goal ^74– 76^, although challenges remain in developing long-term strategies ^77^. Modelling can make useful contributions in developing sustainable, effective interventions and surveillance strategies and should therefore be included in any programmatic design from the start. As embodied by the 2021-2030 NTD roadmap, impactful interventions cannot be achieved by working in silos, but instead require continuous communication between all parties of an interdisciplinary team.

## Drug development and clinical study design

Though modelling is increasingly used in public health decision making, the use of modelling to direct clinical trial design and drug development is not so common, and even less so for NTDs. To reach goals like elimination as a public health programme (trachoma, STH, schistosomiasis and LF) and elimination of transmission (onchocerciasis) novel drug development will be critical ^1, 4, 5, 11, 13, 78^. However, financial returns on investments into NTDs are limited and therefore largely unappealing, particularly because of the heavy reliance by endemic nations, on donations from pharmaceutical producers. Increased use of mathematical modelling could reduce the financial waste associated with the drug-development-to-distribution-pipeline ^78^. If we consider this pipeline in three parts; pre-clinical, clinical trial and distribution, it is clear that modelling can provide valuable insight at each stage. Onchocerciasis and LF have recently benefited from pharmacokinetic-pharmacodynamics modelling, translating pre-clinical non-human experimental results into quantitative insights relevant to human treatment ^79^. Clinical trial simulations are designed to include all aspects of a clinical trial protocol including (but not limited to) recruitment criteria, drug properties/ effectiveness and follow-up times ^80^, providing valuable guidance that translates into more effective, efficient, cost-efficient and robust clinical trials. In addition to providing insight into the optimal distribution of new drugs ^81^, rethinking the distribution of existing drugs to achieve public health targets can also be guided by modelling ^37, 48^.

## Challenges

Modelling has certainly addressed many of the key questions asked of modellers at the 2019 meeting ^2^. However, cross-disease challenges remain ^82^. The most common of these, highlighted by all groups involved in the meeting report ^2^ and this collection, is undoubtedly a lack of data or poor data quality. This could be because certain parameters simply cannot be measured; because of vast heterogeneity or because they have yet to be collected ^83^. For example, VL has a highly variable incubation period, unknown duration of asymptomatic infection and estimates for the duration of lasting immunity are ill-defined ^6, 84, 85^, introducing uncertainty into the temporal dynamics underlying any projections. Chagas disease, gHAT and leprosy also suffer from indeterminate incubation periods ^9, 12, 21^ impacting case detection and adding greater uncertainty in epidemiological estimates fitted to by models ^85, 86^. Asymptomatic or pre-symptomatic infection is common of many NTDs and presents a significant challenge to their management. For example, asymptomatic VL infections cannot be treated, whereas it is possible to teat asymptomatic gHAT but only if it is able to be detected. Identifying their respective proportions in an infected population, particularly in the absence of high surveillance coverage, means accounting for this group using roundabout methods and proxy diagnostics ^6, 9^.

Many diagnostics are indirect, proxy measures of case detection, often with less than perfect sensitivity or specificity ^87, 88^, and have a direct effect on perceived prevalence and individual burdens of infection ^89, 90^. Given that models are only as good as the data to which they are fitted, this has a significant impact on the utility of model results. For example, in the instances of STH and intestinal schistosomiasis ( *Schistosoma mansoni*), WHO targets are given in terms of eggs per gram of faecal matter as detected with the Kato-Katz method, which notoriously suffers from poor sensitivity, particularly for low intensity infections ^91^, invariably underestimating prevalence. Where a multi-host system is present for zoonotic NTDs, though it is possible to measure infection through direct observation of parasite stages in the animal host(s) ^14^, via necropsy or other methods ^92^, it is likely that this approach is inappropriate for monitoring and evaluating the likes of *T. solium* control programmes, due to the large animal sample sizes required to detect a statistically meaningful impact on transmission, especially in low prevalence settings ^14^. Molecular xenomonitoring (testing vectors for the parasite instead of human hosts) for LF and onchocerciasis has shown promise ^93^ but operational research gaps remain, impacting large-scale utilisation ^94^. Reconciling these different streams of imperfect diagnostic data will be key to their utility in modelling and indeed to reaching and sustaining public health goals.

The operational units over which epidemiological data are collected, and projections made are also often over somewhat arbitrary administrative borders that infectious diseases do not adhere to. For rabies, non-spatial models are inadequate for capturing the low-endemicity incidence rates ^15^ such that more data-intensive modelling approaches are required. In addition to questionable detection success, VL surveillance has operated over geographical units that are too large to evaluate the success of control methods ^6^, despite modelling showing that transmission is highly localised over smaller spatial scales (i.e. 85% of inferred transmission distances ≤300m) ^95^. Similarly for onchocerciasis, modelling shows that the rate at which interventions can be scaled down depend strongly on the spatial units of assessment ^13, 96^. Clustering of *T. solium* porcine cysticercosis around human taeniasis carriers, particularly evident in South American communities, demonstrates the need for spatially explicit models in certain settings ^14, 97^, such as the recently developed CystiAgent model for Peru ^98^, capable of testing spatially structured interventions. From this it is evident that whilst spatial heterogeneity requires nuanced model structure, the leading challenge here is the paucity of data at the spatial level necessary to parameterise the models for spatially relevant insights. This will become ever more important as all NTDs move towards low-prevalence and spatially-heterogenous incidence patterns.

The assumptions made to overcome these uncertainties often differ across models – which then produce differing results. Whilst this is somewhat overcome by the practice of model comparison ^99, 100^, which highlights important biological and population processes that impact epidemiological trajectories, these unknowns wave a clear flag for collaborative opportunities between modellers, field epidemiologists and clinicians. Indeed, the optimal working relationship is a synergistic pathway, where the model’s needs drive data collection, the data shapes further model iterations, and these then inform policy and the outcomes at the programmatic and clinical level ^51, 82, 83, 85, 101– 109^. Improving communication between these groups is critical to achieving the desired public health gains ^20^.

## Conclusion

The increased use of mathematical and statistical modelling over the last decade has helped move the field of NTDs into a more quantitative space, providing the link between epidemiological concepts and observed reality. For modelling to continue to fill this role and influence decision-making, ongoing conversations and engagement between all parties will be paramount. These will, in turn, overcome the continuous challenges of data quality and access, and the consequent model assumptions required. As programme and disease management move towards a country-ownership framework under the new roadmap, it will be key that modelling follows suit, overcoming systematic notions of knowledge ownership and challenging associated power dynamics ^110– 112^. In this way, future modelling will work to support this new NTD landscape. 

## Data availability

No data are associated with this article.
