# Supplementary material for: How modelling can help steer the course set by the World Health Organization 2021-2030 roadmap on neglected tropical diseases
Source: Gates Open Res. 2022 Feb 2;5:112. Originally published 2021 Jul 27. [Version 2] doi: 10.12688/gatesopenres.13327.2 (PMC8816801; doi:10.12688/gatesopenres.13327.2)
Supplement: Peer review history

# Peer review: Reviewer response for version 2

**DOI:** 10.21956/gatesopenres.14821.r31726

**Published:** 2022-02-04

**Authors:** Angus McLure

**Affiliations:** 1 National Centre for Epidemiology and Population Health, Australian National University, Acton, ACT, Australia

The changes implemented by the authors have addressed my (minor) reservations.

Does the article adequately reference differing views and opinions?

Partly

Are all factual statements correct, and are statements and arguments made adequately supported by citations?

Partly

Is the Open Letter written in accessible language?

Yes

Where applicable, are recommendations and next steps explained clearly for others to follow?

Yes

Is the rationale for the Open Letter provided in sufficient detail?

Yes

Reviewer Expertise:

Infectious disease modelling broadly, with some experience with LF but not the other NTDs covered by this letter.

I confirm that I have read this submission and believe that I have an appropriate level of expertise to confirm that it is of an acceptable scientific standard.

# Peer review: Reviewer response for version 1

**DOI:** 10.21956/gatesopenres.14570.r31306

**Published:** 2022-01-04

**Authors:** Margaret C. Baker

**Affiliations:** 1 Georgetown University, Washington, DC, USA

This article is an introduction to the series of articles that have been published describing the role of mathematical modelling in the development of the goals and objectives set by the World Health Organization in the 2021-2030 roadmap for NTDs. The article provides a good overview of the series which in turn provides welcome visibility into how mathematical models informed the development of the 2030 roadmap.The article makes the case for use of modeling in regards to informing: 1) Programmatic timelines, 2) Program design and 3) Drug development.  

 With regard to the first 2 items (timelines and program design), the case is clearly made for the potential of modeling to inform these, at least at a global level. What is less clear is how modeling can inform decision making at a national level to answer similar questions. This would require the use of an interactive tool that can be used by NTD program managers and their partners to input their parameters (e.g. disease agent, vector species, baseline prevalence, different intervention details, etc). They would in turn be provided with predictions on timelines and answers to questions such as: what would be the impact of: increasing coverage? Reducing those never treated by x %? Adding complimentary control methods? Increasing frequency of treatment? Such tools are available for other health areas like maternal and neonatal health (e.g http://www.healthpolicyplus.com/ns/pubs/18466-18842_MHTools.pdf; ​​ http://www.mandate4mnh.org/;

 Jones-Hepler *et al.*, 2017 ^1^)

 With regards to drug development I am not aware of the need for new drugs for LF or trachoma (the references provided did not collaborate and these are not listed as challenges in the Roadmap). 

 As a reviewer I was asked to comment on whether the article adequately referenced different views and opinions, to which I respond, only partially. The article is authored by modellers who have a much clearer insight into the pro modelling perspective. There are still many in the NTD community who remain skeptical - due to one of the challenges that is clearly presented in this article, that is the reliability of the data on which the models are built. It would be helpful to have all the assumptions, parameters, and data sources used in the models published online in one easy-to-access place. This would enable more informed discussions to take place, across a wider group of participants, as existing evidence is weighed in making policy decisions. It would also be helpful to have a summary from this of the biggest evidence gaps - to drive research and documentation of programmatic results.

Does the article adequately reference differing views and opinions?

Partly

Are all factual statements correct, and are statements and arguments made adequately supported by citations?

Partly

Is the Open Letter written in accessible language?

Yes

Where applicable, are recommendations and next steps explained clearly for others to follow?

Not applicable

Is the rationale for the Open Letter provided in sufficient detail?

Yes

Reviewer Expertise:

PC NTDs, implementation science, design and evaluation of infectious disease control programs

I confirm that I have read this submission and believe that I have an appropriate level of expertise to confirm that it is of an acceptable scientific standard.

## References

[1] : Maternal and Neonatal Directed Assessment of Technologies (MANDATE): Methods and Assumptions for a Predictive Model for Maternal, Fetal, and Neonatal Mortality Interventions. *Global Health: Science and Practice* .2017;5(4) : 10.9745/GHSP-D-16-00174 571-580 10.9745/GHSP-D-16-00174 PMC575260429284695

# Response

**Published:** 2022-01-28

**Authors:** Jessica Clark

_*What is less clear is how modeling can inform decision making at a national level to answer similar questions. This would require the use of an interactive tool that can be used by NTD program managers and their partners to input their parameters (e.g. disease agent, vector species, baseline prevalence, different intervention details, etc). They would in turn be provided with predictions on timelines and answers to questions such as: what would be the impact of: increasing coverage? Reducing those never treated by x %? Adding complimentary control methods? Increasing frequency of treatment? Such tools are available for other health areas like maternal and neonatal health (e.g http://www.healthpolicyplus.com/ns/pubs/18466-18842_MHTools.pdf; ​​ http://www.mandate4mnh.org/;*_

_*Jones-Hepler et al., 2017 ^1^ )*_

**A similar point has been raised by reviewer 1. A new final paragraph of the C *hallenges *section has been added. In short, the translation of international-level policy to local-level implementation does mean that local-level specific details are often overlooked, and general policy recommendations fail to meet all conditions of that setting. Whilst providing tools locally is an obvious path to improve national (or smaller) level control, it comes with a trade-off. In making the models more user-friendly, some of the complexities that allow for fine scale tuning of certain parameters may have to become fixed, such that the insight is less specific. There is also the matter of interpretation and understandings the limitations of the models. As such, local expertise is still necessary even in the hands of decision-makers.**

_*With regards to drug development I am not aware of the need for new drugs for LF or trachoma (the references provided did not collaborate and these are not listed as challenges in the Roadmap).*_

**This misunderstanding was a result of poor sentence structure. We have rectified this by rewriting the opening to the *Drug development and clinical study design *section.**

****

_*As a reviewer I was asked to comment on whether the article adequately referenced different views and opinions, to which I respond, only partially. The article is authored by modellers who have a much clearer insight into the pro modelling perspective. There are still many in the NTD community who remain skeptical - due to one of the challenges that is clearly presented in this article, that is the reliability of the data on which the models are built. It would be helpful to have all the assumptions, parameters, and data sources used in the models published online in one easy-to-access place. This would enable more informed discussions to take place, across a wider group of participants, as existing evidence is weighed in making policy decisions. It would also be helpful to have a summary from this of the biggest evidence gaps - to drive research and documentation of programmatic results.*_

**It is out with the scope of this article to provide a thorough overview of the data needs to improve modelling insights. There is however a PLoS NTD collection that was cited in the main text. To make this clearer, text has been added text to the *Challenges *section, directing the reader to this in-depth collection. With regards to the assumptions, parameters and data sources; these are provided in every publication that presents modelling results, and the code for each model is public. These are not included here because there are no models specifically presented here.**

# Peer review: Reviewer response for version 1

**DOI:** 10.21956/gatesopenres.14570.r31527

**Published:** 2021-12-14

**Authors:** Angus McLure

**Affiliations:** 1 National Centre for Epidemiology and Population Health, Australian National University, Acton, ACT, Australia

This open letter summarises the contributions of (infectious) disease modelling to inform elimination efforts for Neglected Tropical Diseases (NTDs), highlighting key areas of contributions (timelines/forecasts/predictions, Programme/intervention/surveillance design, and drug development) and naming some of the key challenges. To be frank, it appears to me the kind of letter that doesn't fit very well into the peer-review paradigm as the goal is primarily to introduce a collection of papers. However, I have tried to answer the peer-review questions as posed and provide constructive feedback where I can. I should note that my relevant area of knowledge is mostly around LF and infectious disease modelling more broadly, so my review is skewed towards topics most relevant to LF.

 Regarding the correctness of all statements, to my knowledge, nearly all the statements are correct. However, I would like to highlight one statement which seemed at odds with the literature. The line comes in paragraph 10: 'To reach goals like elimination as a public health programme (trachoma, STH, schistosomiasis and LF) and elimination of transmission (onchocerciasis) novel drug development will be critical'. With regards to LF, I don't agree with this statement, i.e. I don't think new drug development is critical for the elimination of LF as a public health problem. Moreover the statement appears to contradict the Consortium's recent paper specifically on LF elimination targets (NTD Modelling Consortium Lymphatic Filariasis Group, 2019 ^1^). From my knowledge of LF, the existing drugs (ivermectin, albendazole, and diethylcarbamazine) work well and as long as drugs are matched to the setting, side effects are not major concerns. The Consortium's existing work suggests that the 2030 goals can be achieved with these existing drugs if programmes can effectively deliver enough rounds of MDA before 2030. The main barrier to this elimination campaign is achieving high coverage (with the existing drugs) and reaching areas or people who have consistently low treatment coverage. Even a 100% effective and safe medicine won't help if some people or foci aren't being treated. Whether this criticism can be extended to the other diseases mentioned here is beyond my knowledge. This is the only point in the paper I have real reservations about.

 I have indicated in my review that the letter only *partly* references differing views and opinions. Though I don't think this represents a major defect of the letter, I should explain why I chose this response. As the main thrust of the letter is that modelling has been and can continue to be useful for directing elimination efforts, presenting differing views and opinions would involve finding examples of where models have *mis*directed elimination efforts or sharing the opinions those who hold that 'models are a waste of time'. While I have certainly come across those who hold the latter view, they tend not to express their view in the form of citable literature, so the absence of their opinions is perhaps to be expected! However, as regards instances where models have misdirected elimination efforts, given the large volume of literature covered, there are probably examples of this — i.e. where a model recommended an intervention that turned out to be far from adequate. Whether these examples are publicly available and known to the authors is another question. If they know of good examples, the authors might consider including these, perhaps with a note about how these failures have informed future modelling work.

 Having said all this, I think the 'challenges' section of the paper covers the major challenges reasonably enough. This section can perhaps be improved by giving more examples of how these challenges translate into things that models *cannot* or have not yet been able to do; currently the section feels a little too modelling-centric rather than programme-centric. An example of something models have not been able to do (that I am aware of) is around critical prevalence thresholds for LF/oncho elimination. Various models investigating these have found that the critical threshold varies substantially based on setting-specific assumptions. However, knowing that the threshold will be different in different settings doesn't really help you pick a target threshold that's appropriate for *your* setting. And since there haven't been resources in place to fit models to every setting, we have ended up with the very crude 1/2% prevalence thresholds which will be unnecessarily low in some settings and too high in others (e.g. places with high and highly heterogenous biting rates). This to me is an example not of the failure of *modellers*, but a limitation of modelling as an approach to help inform *specific* interventions/programmes. You might argue that the 1%/2% thresholds are targets not *necessarily* meant to be identified with critical transmission tipping points. However my reading of the non-modelling literature and conversations with non-modellers suggests that many people believe that the 1%/2% target thresholds entail the interruption of transmission and that many believe that the universal application of these thresholds have a rigorous evidence base. Moreover, discussion of resurgence often blame failure to achieve the threshold rather than suggesting that the threshold might be wrong for the setting. Again this perhaps points to a failure of communication rather than a failure of the modelling itself, but as this letter is about how modelling informs practice, this example (or better example along a similar line) may be relevant for inclusion (if they can manage to express it more succinctly than I have!).

 A final minor point:

 P10. Misplaced comma in "by endemic nations, on donations".

Does the article adequately reference differing views and opinions?

Partly

Are all factual statements correct, and are statements and arguments made adequately supported by citations?

Partly

Is the Open Letter written in accessible language?

Yes

Where applicable, are recommendations and next steps explained clearly for others to follow?

Yes

Is the rationale for the Open Letter provided in sufficient detail?

Yes

Reviewer Expertise:

Infectious disease modelling broadly, with some experience with LF but not the other NTDs covered by this letter.

I confirm that I have read this submission and believe that I have an appropriate level of expertise to confirm that it is of an acceptable scientific standard, however I have significant reservations, as outlined above.

## References

[1] : The roadmap towards elimination of lymphatic filariasis by 2030: insights from quantitative and mathematical modelling. *Gates Open Res* .2019;3: 10.12688/gatesopenres.13065.1 1538 10.12688/gatesopenres.13065.1 31728440PMC6833911

# Response

**Published:** 2022-01-28

**Authors:** Jessica Clark

_*Regarding the correctness of all statements, to my knowledge, nearly all the statements are correct. However, I would like to highlight one statement which seemed at odds with the literature. The line comes in paragraph 10: 'To reach goals like elimination as a public health programme (trachoma, STH, schistosomiasis and LF) and elimination of transmission (onchocerciasis) novel drug development will be critical'. With regards to LF, I don't agree with this statement, i.e. I don't think new drug development is critical for the elimination of LF as a public health problem. Moreover the statement appears to contradict the Consortium's recent paper specifically on LF elimination targets (NTD Modelling Consortium Lymphatic Filariasis Group, 2019 ^1^ ). From my knowledge of LF, the existing drugs (ivermectin, albendazole, and diethylcarbamazine) work well and as long as drugs are matched to the setting, side effects are not major concerns. The Consortium's existing work suggests that the 2030 goals can be achieved with these existing drugs if programmes can effectively deliver enough rounds of MDA before 2030. The main barrier to this elimination campaign is achieving high coverage (with the existing drugs) and reaching areas or people who have consistently low treatment coverage. Even a 100% effective and safe medicine won't help if some people or foci aren't being treated. Whether this criticism can be extended to the other diseases mentioned here is beyond my knowledge. This is the only point in the paper I have real reservations about.*_

**The reference to LF was with regards to the fact that it has been targeted for elimination as a public health problem, rather than the need for new therapeutics specifically for LF. However, we acknowledge that the sentence was misleading and have rewritten the opening to the *Drug development and clinical study design *section.**

*_I have indicated in my review that the letter only partly references differing views and opinions. Though I don't think this represents a major defect of the letter, I should explain why I chose this response. As the main thrust of the letter is that modelling has been and can continue to be useful for directing elimination efforts, presenting differing views and opinions would involve finding examples of where models have misdirected elimination efforts or sharing the opinions those who hold that 'models are a waste of time'. While I have certainly come across those who hold the latter view, they tend not to express their view in the form of citable literature, so the absence of their opinions is perhaps to be expected! However, as regards instances where models have misdirected elimination efforts, given the large volume of literature covered, there are probably examples of this — i.e. where a model recommended an intervention that turned out to be far from adequate. Whether these examples are publicly available and known to the authors is another question. If they know of good examples, the authors might consider including these, perhaps with a note about how these failures have informed future modelling work._*

**The second paragraph of *Programme design*highlighted some of the ways in which inherent features of the target populations (largely related to behaviour – adherence, treatment access etc) can impact the model projections. This has been made more explicit with the addition of a specific example regarding VL.**

_*Having said all this, I think the 'challenges' section of the paper covers the major challenges reasonably enough. This section can perhaps be improved by giving more examples of how these challenges translate into things that models cannot or have not yet been able to do; currently the section feels a little too modelling-centric rather than programme-centric. An example of something models have not been able to do (that I am aware of) is around critical prevalence thresholds for LF/oncho elimination. Various models investigating these have found that the critical threshold varies substantially based on setting-specific assumptions. However, knowing that the threshold will be different in different settings doesn't really help you pick a target threshold that's appropriate for your setting. And since there haven't been resources in place to fit models to every setting, we have ended up with the very crude 1/2% prevalence thresholds which will be unnecessarily low in some settings and too high in others (e.g. places with high and highly heterogenous biting rates). This to me is an example not of the failure of modellers, but a limitation of modelling as an approach to help inform specific interventions/programmes. You might argue that the 1%/2% thresholds are targets not necessarily meant to be identified with critical transmission tipping points. However my reading of the non-modelling literature and conversations with non-modellers suggests that many people believe that the 1%/2% target thresholds entail the interruption of transmission and that many believe that the universal application of these thresholds have a rigorous evidence base. Moreover, discussion of resurgence often blame failure to achieve the threshold rather than suggesting that the threshold might be wrong for the setting. Again this perhaps points to a failure of communication rather than a failure of the modelling itself, but as this letter is about how modelling informs practice, this example (or better example along a similar line) may be relevant for inclusion (if they can manage to express it more succinctly than I have!).*_

**This is an interesting point and, if we have distilled it correctly, we completely agree. What you describe is perhaps not so much a failure of modelling, or something models cannot do, but is a symptom of the framework in which we apply mathematical modelling to NTDs. Large-scale international-level policy and guidance is the outcome of mathematical models that are indeed fit to data from specific locations, likely using more than one model, however it is unlikely that this insight will adequately describe all settings. So, there is a disparity across the public health hierarchy from international-level modelling use to a local level. The point you raise about non-modellers then using certain values as wrote is, as you rightfully state, an outcome of communication and understanding. This is a nuanced point connected to local access to, and use of, models. Reviewer two has raised the point that a discussion should be had in this letter around the topic of making models available as tools. These two comments are not mutually exclusive. We have therefore addressed them simultaneously in an new final paragraph of the *Challenges *section.**

_*A final minor point:*_

_*P10. Misplaced comma in "by endemic nations, on donations".*_

**This has been rectified, thank you.**
